# Clinical Uses of Melatonin in Pediatrics

**DOI:** 10.1155/2011/892624

**Published:** 2011-06-16

**Authors:** Emilio J. Sánchez-Barceló, Maria D. Mediavilla, Russel J. Reiter

**Affiliations:** ^1^Department of Physiology and Pharmacology, School of Medicine, University of Cantabria and Institute of Formation and Research “Marques de Valdecilla” (IFIMAV), 39011 Santander, Spain; ^2^Department of Cellular and Structural Biology, University of Texas Health Science Centre, San Antonio, TX 78229, USA

## Abstract

This study analyzes the results of clinical trials of treatments with melatonin conducted in children, mostly focused on sleep disorders of different origin. Melatonin is beneficial not only in the treatment of dyssomnias, especially delayed sleep phase syndrome, but also on sleep disorders present in children with attention-deficit hyperactivity, autism spectrum disorders, and, in general, in all sleep disturbances associated with mental, neurologic, or other medical disorders. Sedative properties of melatonin have been used in diagnostic situations requiring sedation or as a premedicant in children undergoing anesthetic procedures. Epilepsy and febrile seizures are also susceptible to treatment with melatonin, alone or associated with conventional antiepileptic drugs. Melatonin has been also used to prevent the progression in some cases of adolescent idiopathic scoliosis. In newborns, and particularly those delivered preterm, melatonin has been used to reduce oxidative stress associated with sepsis, asphyxia, respiratory distress, or surgical stress. Finally, the administration of melatonin, melatonin analogues, or melatonin precursors to the infants through the breast-feeding, or by milk formula adapted for day and night, improves their nocturnal sleep. Side effects of melatonin treatments in children have not been reported. Although the above-described results are promising, specific studies to resolve the problem of dosage, formulations, and length of treatment are necessary.

## 1. Introduction

Since the identification and isolation of melatonin from bovine pineals [1], especially in the last 20 years, numerous clinical trials were designed to study the possible therapeutic usefulness of this pineal indoleamine in different fields of medicine [2]. The objective of this paper is to review, in depth, the state of the art on the clinical uses of melatonin in pediatrics. Although, at present, many experimental studies are providing the basis for future clinical applications of melatonin in different pediatric pathologies (i.e., obesity, enuresis, etc.), this paper focuses only on those medical applications which have been already assessed in clinical trials. Some of the basic aspects on the usefulness of melatonin in this clinical area have been recently reviewed by Gitto et al. [3]; the readers are also referred to this paper. 

Among the wide spectrum of properties attributed to melatonin, perhaps one of the most solidly based is its regulation of the sleep/wake cycle [4]. Thus, it is not surprising that most clinical applications of melatonin in pediatrics are focused on childhood sleep disorders of different origin but especially of circadian etiology. Also, when melatonin was used for this purpose in children, it is noteworthy that significant side effects of the indoleamine have not been reported [5].

## 2. Melatonin Uses for Children's Sleep Disorders

Among elementary school children, bedtime resistance is the most prevalent sleep problem (27%). Sleep-onset delays (11.3%), night waking (6.5%), morning wakeup problems (17%), and fatigue complaints (17%) are also common [6]. Although sleep problems of different etiology are the frequent cause of medical consults, there are no specific prescription drugs for the treatment of insomnia in children, and many pediatricians recommended behavioural treatments in an attempt to correct sleep problems in infants. However, melatonin is being prescribed as a medication associated to behavioural treatment for sleep disorders by an increasing number of physicians, judging from a survey carried out by Owens et al. [7]. 

The hypothesis of the relationship between melatonin and sleep was founded on the coincidence of highest circulating levels of melatonin and depressed core body temperature [8] with the greatest nocturnal sleepiness [9]. Furthermore, in some individuals, the administration of physiologic doses (0.1–0.3 mg) of melatonin promotes sleep onset and maintenance, decreases sleep latency, increases sleep efficiency, and improves total sleep time [10, 11]. A review of the basic mechanism involved in melatonin's sleep regulation can be found in a publication by Dubocovich [12].

The American Academy of Sleep Medicine, in The International Classification of Sleep Disorders (ICSD) [13], defines four categories: (1) dyssomnias, (2) parasomnias, (3) sleep disorders associated with mental, neurologic, or other mental disorders, and (4) proposed sleep disorders. In this paper, we consider the possible usefulness of melatonin in children in some sleep disorders included in groups 1 and 3 of this classification.

### 2.1. Dyssomnias

The ICSD defines dyssomnias as “the disorders that produce either difficulty initiating or maintaining sleep.” This section includes the circadian rhythm sleep disorders (those related to the timing of sleep during the 24-hour day) and, among them, delayed sleep-phase syndrome (DSPS) and advanced sleep-phase syndrome (ASPS). The DSPS is a disorder in which the major sleep episode is delayed in relation to the desired clock time, resulting in symptoms of sleep onset or difficulty in awakening at the desired time [13]. Typically, patients with DSPS fall asleep several hours after midnight and have difficulty waking up in the morning [14]. In the ASPS, the problem is that the major sleep episode is advanced in relation to the desired clock time, and the patients exhibit an early sleep onset (evening sleepiness) and an early awakening. While ASPS is very rare among children but frequently encountered in the elderly and in postmenopausal women, DSPS usually develops in early childhood or adolescence, and it is more prevalent among younger people [14]. The time of melatonin secretion onset in DSPS patients is significantly delayed compared with healthy controls [15].

Several clinical trials have evaluated the efficacy of melatonin as a therapy for DSPS in adults [16]. In children, only a retrospective study [17] describes the effects of long-term treatment with melatonin (3–5 mg/day for an average period of 6 months) in 33 adolescents with DSPS. This treatment advanced the sleep onset and increased sleep duration and was also associated with a reduction in the proportion of patients reporting school difficulties [17]. There also is a relationship between timing of melatonin administration and phase changes in patients with DSPS. Thus, in a double-blind placebo-controlled trial carried out on 13 subjects with DSPS, melatonin (0.3 or 3.0 mg) or placebo was administered for a 4-week period, between 1.5 and 6.5 hours prior to DLMO (dim light melatonin onset). DLMO, the time of the evening melatonin rise under dim light environment [18], is an excellent marker of circadian phase of endogenous melatonin which can be measured in the awake patient, before sleep time; the DLMO provides valuable information about the phase of the circadian pacemaker [15, 19]. The magnitude of phase advance in DLMO and the advance in sleep time were higher at earlier times of melatonin administration [20]. Recently, a randomized, placebo-controlled double-blind trial carried out on 72 children with chronic sleep onset insomnia, treated during a week with either melatonin (0.05, 0.1, or 0.15 mg/kg) or placebo, reported even with the lowest doses a significant (1 h) advance of the sleep onset and DLMO as well as a shortening of sleep latency [21]. These effects of melatonin are greatest when treatment is administered at least 1-2 h before DLMO [21]. In a review on circadian rhythm sleep disorders, sponsored by the American Academy of Sleep, Sack et al. [22] concluded that “The evidence is quite strong that melatonin, timed to promote a corrective phase advance, is an effective treatment for DSPS”; this suggests additional studies to determine the optimal parameters for dosing and scheduling. For practical reasons, since determination of DLMO is not a usual clinical procedure, it is good practice to administer melatonin just before the desired bedtime. When melatonin is given 30 minutes before bedtime, the hypnotic, more than the chronobiotic, effects of the indoleamine could potentially ameliorate the sleep-onset difficulties in patients with DSPS [20, 21].

### 2.2. Sleep Disturbances Associated with Mental, Neurologic, or Other Medical Disorders

Irregular patterns of sleep-wake rhythm are commonly associated with neurological impairment [23], and the possible therapeutic value of melatonin for these sleep disorders has been assessed in several clinical trials [24]. The neurological diseases that are associated with sleep disorders included mental or intellectual disability, mental retardation, learning disabilities, autistic spectrum disorders, Rett syndrome, tuberous sclerosis, developmental disabilities, and Angelman syndrome. A recent meta-analysis [25] of data from 9 randomised placebo-controlled trials published between 1990 and 2008 comprising a sample of 183 patients concluded that melatonin, at doses ranging from 0.5 to 9 mg, decreases sleep latency, increases total sleep time, and reduces the number of awakenings per night in individuals with intellectual disabilities. Other clinical trials not included in the above-mentioned meta-analysis showed similar results. Thus, Jan et al., [26] in a pioneering study in this field, noted that in 15 children with different neurological problems associated with severe chronic sleep disorders unresponsive to conventional management, when given 2 to 10 mg of oral melatonin, a significant improvement of their sleep and behavioral problems was apparent. Similarly, Zhdanova et al. [27] reported that 0.3 mg melatonin improved the objective and subjective sleep quality of children with Angelman syndrome, while Pillar et al. [28], in five children (mean age 8.2 ± 3.6 years) with severe psychomotor retardation and with irregular sleep-wake patterns, reported that melatonin treatment (3 mg/day) increased significantly nighttime sleep and sleep efficiency and reduced daytime sleep [28].

Not only blind children with other neurologic pathologies but also those suffering only visual impairments often have associated sleep-wake cycle disorders [29]. Several case reports [30–32] as well as an open study in 8 children and young adults [33] recommended the treatment of these sleep disorders with melatonin. Most totally blind children, in the absence of an entraining light-dark cycle, develop free running sleep/wake rhythms [34, 35]. These children may benefit from therapy with melatonin. 

Among the mental and neurological disorders with associated sleep problems, several pathologies have been especially considered for treatment with melatonin and deserve particular emphasis. This includes autism spectrum disorders and attention-deficit hyperactivity disorder. Smith-Magenis and Sanfilippo syndromes, pathologies also associated with sleep disturbances susceptible of treatment with melatonin, will be also analyzed in depth.

### 2.3. Autism Spectrum Disorders (ASDs)

Insomnia is the predominant sleep concern in children with ASD, and its nature is most likely multifactorial including several neurochemical etiologies (e.g., abnormalities in serotonergic transmission or melatonin levels). The etiologic role of melatonin in sleep problems associated with ASD has been suggested in numerous studies [36, 37]. Children with ASD show lower melatonin levels than healthy children [38], possibly caused by a primary deficit in one or both of the enzymes, that is, AA-NAT (aralkylamine N-acetyltransferase) and ASMT (acetylserotonin methyltransferase, also known as HIOMT: hydroxyindole O-methyltransferase), which are responsible for the conversion of N-acetylserotonin to melatonin. The gene coding for ASMT enzyme is frequently mutated in ASD pat [39, 40]. Possible mutations in genes coding for melatonin receptors in patients with ASD also have been studied although without conclusive results [41].

Melatonin has been assessed for its possible usefulness in the treatment of sleep troubles of ASD patients for more than 15 years (see the most recent reviews, [42, 43]). In a study of 15 children (6–17 years) with Asperger disorder, a form of ASD, treated with melatonin (3 mg) for 14 days, sleep and behavioral improvements were observed in response to melatonin [44]. These were confirmed in a randomized, placebo-controlled double-blind crossover trial recruiting 11 children with ASD [45]. These children received either melatonin (5 mg) or placebo for a period of 4-weeks; this was followed by a washout period of 1 week followed by a second 4-week treatment period. Although only 7 children completed the trial, and this small sample diminished the significance of the results, the conclusion was that all the outcome parameters (sleep latency, awakenings per night, and total sleep duration) improved with melatonin treatment. More interesting is the study carried out by Giannotti et al., [46] in 25 children in which the melatonin formulation (3 mg) consisted of 1 mg fast release and 2 mg controlled release, which was given in long term (12–24 months). Children's Sleep Habits Questionnaire and sleep diaries were used to assess sleep quality before and after treatment and after discontinuation. The administration of melatonin improved sleep parameters in all cases; 64% of patients returned to pretreatment scores after discontinuation of melatonin, but they responded again positively to the restoration of the treatment. Controlled-release melatonin (5 mg) was also used in another randomized double-blind placebo-controlled crossover trial in a group of 51 children with neurodevelopmental disabilities, 16 of whom had been diagnosed with ASD [47]. Melatonin treatment improved the sleep of 47 of these children although the results were globally analyzed without distinguishing the different types of neurodevelopmental pathologies included in the study (cerebral palsy, epilepsy, visual impairment, etc.). An even larger patient sample was studied by [48]. These authors carried out a clinical trial on 107 children (2–18 years of age) with a confirmed diagnosis of ASD, treated with melatonin (0.75–6 mg). In 25% of these individuals, based on the parent's reports, sleep troubles disappeared; in most cases (60%), at least a significant improvement of sleep was reported, although some problems persisted, 14 children did not experience changes in their sleep quality; and, in one patient, sleep worsened. The conclusion was that melatonin appears to be an effective, safe, and a well-tolerated treatment for insomnia in children with ASD. New studies have confirmed these results. Thus, a 4-week, randomized, double-blind, placebo-controlled crossover was performed in which either melatonin (3 mg) or placebo was given to participants for 2 weeks; the treatments were then crossed over for another 2 weeks. The results of this study also support the efficacy and tolerability of melatonin treatment for sleep problems in children with ASD [49]. A more recently published study is double-blind, randomized, controlled crossover trial involving 22 children diagnosed of ADS, with severe dyssomnias refractory to supportive behavioral management. Patients were treated for 3 months with placebo versus 3 months of melatonin (maximum dose of 10 mg). In all children (17 cases) who completed the study, melatonin significantly improved sleep latency and total sleep time compared to placebo, but did not influence the number of night awakenings [50]. Interestingly, also in adults with ASD, a retrospective study reported that melatonin (3 mg at bedtime) appears to be effective in reducing sleep onset latency and is probably beneficial in improving nocturnal awakenings and total sleep time [51].

### 2.4. Attention-Deficit Hyperactivity Disorder (ADHD)

Sleep problems including delayed sleep onset, sleep or bedtime resistance, prolonged tiredness upon waking, and daytime sleepiness have been reported in 25–50% of children with ADHD [52]. Two well-controlled studies have focused on the possible role of melatonin in the treatment of sleep disorders in ADHD children. The first was a double-blind, placebo-controlled, 30-day crossover trial carried out in 27 stimulant-treated children (6–14 years of age) with ADHD. Nonresponders to sleep hygiene guidelines were treated with melatonin (5 mg/day, 20 minutes before bedtime) or placebo. Patients receiving melatonin experienced a significant reduction in sleep-onset latency compared to those treated with placebo although the best results were obtained with the combined sleep hygiene and melatonin intervention [53]. The second study, another randomized, double-blind, placebo-controlled trial, recruited 105 medication-free children (6 to 12 years old), with diagnosed ADHD and chronic sleep onset insomnia. Participants were treated with melatonin (3–6 mg, depending on body weight), or placebo for 4 weeks. Melatonin induced a significant advance of sleep onset as well as of the total time asleep as compared to placebo although there was no significant effect on behavior, cognition, or quality of life. Significant adverse events did not occur [54]. The minor adverse effects with melatonin treatment did not significantly differ from placebo in either of these two studies. More recently, Hoebert et al. [55] evaluated the effectiveness and safety of long-term (mean time up to 3.7 years) melatonin treatment in children with ADHD and chronic sleep onset insomnia (CSOI) using questionnaires answered by parents of patients. Interestingly, long-term melatonin treatment was judged to be effective against sleep problems in 88% of the cases; improvement of behaviour and mood was reported in 71% and 61%, respectively, whereas no serious adverse effects were reported. The last and most complete review of the clinical studies on the efficacy and safety of melatonin for the treatment of insomnia in children with ADHD concluded that this indoleamine is a well-tolerated and efficient therapeutic option for these pediatric patients [56].

### 2.5. Smith-Magenis Syndrome (SMS) and Sanfilippo Syndrome (SFS)

SMS is a multiple congenital anomaly common due to a 3.5 Mb interstitial deletion of chromosome 17 band p11.2 and characterized by subtle minor craniofacial anomalies, infantile hypotonia, skeletal findings (brachydactyly, short stature, and scoliosis), developmental and expressive language delays, mental retardation, maladaptive and self-injurious behaviors, and sleep disturbances [57, 58]. Special consideration deserve the sleep disorders of SMS patients which include early sleep onset, difficulty falling asleep, difficulty staying asleep, frequent awakening, early waking, reduced REM sleep, and decreased sleep time [59]. These sleep problems are, in part, responsible for the behavioural and cognitive problems of these children. A high prevalence of sleep disorders, with irregular sleep episodes of different duration randomly distributed over the 24-hour period, has also been described in children with SFS, a type III mucopoysaccharidosis [60].

Whereas the rhythm of melatonin secretion in all mammals, including humans, exhibits a characteristic nocturnal increase in plasma concentration, with lower values during the daytime, patients with SMS have a characteristic inversion of the circadian rhythm of melatonin, with a phase advance shift of 9.6 ± 0.9 h with respect to control subjects [59, 61]. SFS patients also exhibit alterations of the diurnal rhythm of melatonin secretion, with lower concentrations of urinary excretion of 6-sulphatoxymelatonin at night and higher in the morning as compared with healthy controls [62]. 

Since the nocturnal rise of melatonin has been related to the induction of sleep [10], the anomalous secretory pattern of melatonin in SMS and SFS children could, at least in part, be responsible for their sleep disorders. On this basis, a treatment consisting on the administration of a *β*1-adrenergic antagonist in the morning (to abolish the daytime secretion of melatonin) combined with the administration of melatonin in the evening (to generate a nocturnal peak, restoring the normal day/night rhythm of melatonin), has been assessed in SMS children. The result was a dramatic improvement in the sleep quality, evaluated not only by actimetric and subjective methods [59, 63] but with polysomnographic studies [64]. These children experience a better sleep quality, reduced irritability, low evening drowsiness, and improved learning capability. In regard to SFS, two surveys, one among clinicians [65] and another among parents of affected children [66], reported that melatonin is the medication most likely to be of benefit for sleep disorders in these patients. However, clinical trials of case reports with objective measures of sleep quality before and after melatonin treatment have not been published.

## 3. Melatonin Uses in Pediatric Anesthesia

The sedative, anxiolytic, anti-inflammatory, and hypnotic effects of melatonin [67] support the possible use of melatonin at different stages of anaesthetic procedures, from premedication to induction of general anaesthesia or postsurgical analgesia [68]. 

One of the proposed uses of melatonin is premedication preceding the anesthesia induction. The use of melatonin versus midazolam (Dormicum) as a premedicant in children was studied in a randomized, double-blind, placebo-controlled trial involving seven groups of 15 children who receive one of the following premedicants: midazolam (0.1, 0.25, or 0.5 mg/kg), melatonin (0.1, 0.25, or 0.5 mg/kg), or placebo. Premedication with melatonin or midazolam was equally effective in alleviating anxiety although the use of melatonin was associated with a lower incidence of excitement at 10 min postoperatively, and a lower incidence of sleep disturbance at week 2 postoperatively than that observed with midazolam [69]. Other studies reported that midazolam (0.5 mg/kg) is more effective than melatonin (0.05–0.4 mg/kg) in reducing children anxiety in children although the individuals who received melatonin developed less emergence of delirium compared to those who received midazolam [70]. However, another comparative study of melatonin (3 mg, 0.50 mg/kg BW, or 0.75 mg/kg BW, 60 min before procedure) versus midazolam (15 mg) or placebo was carried out on 60 children undergoing sedation for dental treatments. This study reported that melatonin was similar to placebo not contributing to sedation of anxious children [71].

Several diagnostic studies on children require sedation or general anesthesia. One example is brainstem audiometry, an important investigative tool in pediatric audiology. In a survey on 250 children (142 male and 108 female) who underwent auditory brainstem response tests, melatonin-induced sleep allowed for the completion of the exploration in 74–87% of cases [72]. The use of melatonin, especially in children younger than 3 years, has decreased significantly the number of youngsters undergoing general anesthesia for this diagnostic exploration [72]. Magnetic resonance imaging (MRI) examination also requires sedation or general anesthesia to ensure immobility in children who are uncooperative. Forty children undergoing an MRI examination received melatonin (10 mg) 30 min before the scheduled MRI. A subgroup of these children was sleep deprived the night before the exploration. The authors concluded that melatonin, especially if associated with sleep deprivation, improved the success rate of this diagnostic procedure [73]. However, in a stratified randomized double-blind study in children, carried out on 98 patients treated with either melatonin (3–6 mg) or placebo 10 min before they were sedated with chloral hydrate or temazepam plus droperidol for an MRI exam, melatonin did not contribute to sedation [74].

## 4. Melatonin Uses in Epilepsy and Febrile Seizures

The fact that pinealectomy induced violent convulsions in parathyroidectomiced rats as well as observations on the epileptogenic effects of melatonin antibodies intraventricularly injected into rats [75, 76] was the first data suggesting a possible relationship between melatonin and epilepsy. In this context, experimental studies demonstrate that suppression of melatonin, by pinealectomy, increased the brain damage after kainic acid-induced seizures in rats, thus suggesting a neuroprotective role of melatonin [77] on neural tissue. Melatonin was reduced in patients with epilepsy at baseline compared with controls and increased threefold following seizures [78]. Other authors have also reported that patients with seizures of diverse origins show an alteration of the melatonin rhythm [79, 80]. Recent studies, carried out in children with refractory epilepsy or febrile seizures [81, 82], revealed a lowered level of melatonin in these children in comparison with those without seizures. Melatonin could control convulsive crises by acting on both *γ*-aminobutyric acid (GABA) and glutamate receptors [79, 80]. 

The first trials on the possible usefulness of melatonin in epilepsy were carried out on patients refractory to conventional therapies. Although melatonin alone, at a single evening dose of 5–10 mg, has been reported to reduce the frequency of epileptic attacks in children [83], most studies have been focused on the use of melatonin associated with other conventional antiepileptic drugs (vigabatrin, valproate, phenobarbital, etc.). One of the first case reports was concerned with a young girl diagnosed of severe myoclonic epilepsy, with convulsive seizures since her first month of age; melatonin was added to the conventional treatment (Phenobarbital) when the patient was in a precomatose stage, at the age of 29 months. After one month of this therapy and for a year thereafter, the child's seizures were under control [84]. In another study [85, 86] on children with severe intractable seizures, a 3-month treatment with oral melatonin (3 mg/day, 30 min before bedtime) associated with a conventional antiepileptic drug achieved a significant clinical improvement in seizure activity during treatment, particularly during the night. 

The only negative results were reported in a trial on a sample of six children treated with melatonin (5 mg at bedtime). Although melatonin had a positive effect on the patient's sleep disorder, four of six children had elevated seizure activity after treatment [87]. This study had, however, serious weaknesses. One major factor is the small number and heterogeneity of patients: 6 cases with ages ranging from 9 months to 18 years; the comparison between neonatal, infant, or juvenile response to the same doses of melatonin seems inappropriate. Furthermore, the neurological lesions of the six patients are also markedly different. Moreover, the hypothesis of the proconvulsant effects of melatonin has never been confirmed in subsequent studies. Other authors [88] also found that melatonin can be helpful for sleep disturbance in young people with significant neurological impairment although did not find a demonstrable influence of the indoleamine on seizure control.

Since melatonin plays a protective role against the oxidative stress and prevents neuronal damage associated with epilepsy, several clinical trials were focused on the evaluation of the changes in the oxidative status of the patients treated with melatonin alone or associated with other anticonvulsive drugs. In relation to this, 31 children with epilepsy receiving carbamazepine monotherapy [89] and 31 other children treated with valproate [90, 91], who were seizure-free at least for the last 6 months, were involved in a double-blind, randomized, parallel-group, placebo-controlled trial. The effect of add-on melatonin (6–9 mg/day for 14 days) on the antioxidant enzymes glutathione peroxidase and glutathione reductase demonstrated that melatonin is a putative neuroprotector in conditions involving oxidative stress such as that occurs in epilepsies.

Children with epilepsy have high rates of sleep problems due to seizures or anxiety, frequently associated to mental retardation. Twenty-five patients (16 males and 9 females; mean 10.5 years) with these characteristics were randomized to oral fast-release melatonin at bedtime (3 mg/day extended, in case of inefficacy, up to 9 mg/day at 3 mg/week steps) or placebo. Patients treated with melatonin improved their wake-sleep disorders although the seizure frequency was poorly influenced by the treatment [92]. A more recent study, conducted on 23 children with intractable epilepsy treated with oral melatonin before bedtime for 3 months, noticed a significant improvement of both sleep-related phenomena and the severity of seizures in these patients [93].

Although the antiepileptogenic properties of ramelteon, a selective melatonin receptor agonist recently patented, have been described in a rat model of chronic epilepsy [94], clinical trial on its efficiency for human treatment has not been studied.

## 5. Melatonin Uses in Adolescent Idiopathic Scoliosis (AIS)

The hypothesis involving a melatonin deficiency as the source for AIS stems from the fact that experimental pinealectomy in the chicken, rats, rabbits, Atlantic salmon, and mice with genetic deficiency of melatonin forced into a bipedal mode of locomotion results in scoliosis that closely resembles AIS [95, 96].

In humans, Sadat-Ali et al. [97] found serum melatonin levels to be significantly lower in AIS patients than in healthy controls; these results support the hypothesis that serum melatonin levels may contribute to the pathogenesis of AIS. However, these findings were not corroborated by other authors [98–102]. Studies based on the screening of polymorphism in genes coding for melatonin receptors or enzymes involved in melatonin synthesis in AIS patients and controls have generated disparate results [103–106]. A third category of studies, those looking for possible differences in the expression of melatonin receptors in paravertebral muscles on the concave and convex sides of the spinal column in AIS patients, did not uncover conclusive results [107, 108].

A new and interesting approach to the question of the possible role of melatonin in the etiology of AIS is to consider that instead of changes in melatonin production or expression of melatonin receptors, the problem may be an anomalous response of the osteoblast to melatonin in AIS patients [109]. In most cells, melatonin inhibits the forskolin-stimulated adenylyl cyclase activity and decreases cAMP. In contrast, osteoblasts from patients with AIS showed a lack or a marked inhibition by melatonin of the forskolin-stimulated adenylyl cyclase activity [109]. From these findings, a preliminary molecular classification of AIS patients based on the cellular response to melatonin (changes in cAMP) has been proposed [110]. Recently, these authors [110, 111] have also developed the first blood test, based on the cellular reaction to melatonin, to detect children without symptoms who are at risk of developing scoliosis. A prospective analysis on the correlation of serum melatonin levels (monitored yearly for 3–6 years) and curve progression in 40 patients with moderate to severe AIS [112] showed that, from 22 patients with melatonin levels similar to healthy age-matched controls, 16 had stable scoliosis whereas 6 had progressive scoliosis. The 16 patients with low melatonin levels were treated with oral melatonin (3.0 mg 1.5–2.0 hr before the desired sleep time). Twelve of them developed stable scoliosis, whereas four continued to have a progressive course of the disease. This is the first description suggesting that melatonin supplementation may prevent the progression of the scoliosis, especially in mild cases.

## 6. Melatonin Uses in Neonatal Care

Since newborns and particularly those delivered preterm have less protection against oxidation and are highly susceptible to free radical-mediated oxidative damage, melatonin, because of its antioxidant properties, could be useful to reduce oxidative stress in neonates with sepsis, asphyxia, respiratory distress, or surgical stress [113]. These possibilities were exploited in different clinical trials carried out in the Neonatal Intensive Care Unit of the Institute of Medical Pediatrics of the University of Mesina (Italy). 

In one of these studies, 20 newborns with perinatal asphyxia diagnosed within the first 6 hours of life were investigated along with 10 healthy infants. In a random manner, 10 asphyxiated infants received melatonin (80 mg in 8 doses of 10 mg, each separated by 2-hour intervals). Three of the 10 asphyxiated children not given melatonin died within 72 hr after birth; none of the 10 asphyxiated newborns given melatonin died. In the asphyxiated newborns given melatonin, there were significant reductions in malondialdehyde and nitrite/nitrate levels at both 12 and 24 hours, thus indicating the antioxidant effect of melatonin [114]. 

The utility of melatonin as an adjuvant treatment for sepsis was evaluated in a similar randomized clinical trial which included 20 septic newborns; ten of whom received a total of 20 mg of melatonin orally in two doses of 10 mg each, with a 1 h interval, within the first 12 h after diagnosis. Three of 10 septic children who were not treated with melatonin (controls) died within 72 hours after diagnosis of sepsis, whereas none of the 10 septic newborns given melatonin died. Serum levels of lipid peroxidation products, as markers of oxidative stress, in septic newborns treated with melatonin were significantly lower than in placebo-treated infants [115]. 

A third randomized trial [116] recruited 74 newborns with grade III or IV respiratory distress syndrome. Patients received 10 doses of melatonin (10 mg/kg each; 40 patients) or placebo (the remaining 34 cases). Melatonin significantly decreased the severity of respiratory distress syndrome compared to infants receiving placebo.

Finally, the usefulness of melatonin to reduce oxidative stress related to surgical procedures was investigated in 40 newborns with different pathologies subjected to surgical treatment. Ten patients received a total of 10 doses of melatonin (10 mg/kg) at defined intervals for 72 hours by means of intravenous infusion. Ten surgical neonates did not receive melatonin but were given an equal volume of placebo (1 : 50 mixture of ethanol-physiologic saline). Twenty healthy neonates served as control for basal levels of cytokines and nitrite/nitrate. Melatonin reduced postoperative values of cytokines and nitric oxide synthesis in relation to placebo, thus supporting its potent antioxidant properties which resulted in improvement of clinical outcome [117].

## 7. Melatonin Uses in the Feeding of Newborns

It is well documented that melatonin exhibits a circadian rhythm in body fluids, and human milk is no exception. Melatonin in the milk of lactating mothers exhibits a marked daily rhythm, with high levels during the night and undetectable levels during the day [118]. This melatonin rhythm in milk could serve to communicate time of day information to breast-fed infants; this information could also contribute to the consolidation of sleep-wake rhythm of infants until the maturation of their own circadian system occurs. Considering this data, several issues must be considered. First is the importance of nocturnal breast feeding in darkness given that exposure to light causes the suppression of melatonin in breast milk. Second, in milk banks, it may be important to differentiate milk obtained from donors during day versus that collected at night, in order to feed newborns with the milk corresponding to the appropriate periods. Third, for synthetic milk formulations, the composition should differ for night and day products, to achieve a chronobiologic effect on the circadian rhythm in newborns. Thus, it may be worthy to consider the addition of melatonin, melatonin analogues, or melatonin precursors to night milk formulas.

 In regard to this, there is a growing international interest in the development of milk and milk products with a high melatonin contents (patents WO/2001/001784, WO/2007/068361, and US 2008/0058405 A1). In a three-week duration, double-blind trial on 30 infants aged 4–20 weeks with sleeping difficulties, children received “day milk,” with low levels (1.5 gr/100 g protein) of tryptophan, the aminoacid precursor of melatonin, or “night milk,” containing higher levels of tryptophan (3.4 gr/100 g protein) from 06:00 to 18:00 or from 18:00 to 06:00. When the children received the day/night-dissociated milk in concordance with their environment, they showed a significant improvement in the nocturnal sleep parameters that were analyzed (total sleep, sleep efficiency, nocturnal awakenings, and sleep latency) [119]. The urinary metabolites of serotonin on these children suggest that the observed improvements were due to an elevated use of serotonin to melatonin synthesis [120].

## 8. Concluding Remarks

Melatonin, based on its properties as chronobiotic (sleep modulation), antioxidant, or analgesic, all of which have been confirmed in numerous experimental studies, offers perspectives of beneficial effects in a variety of pediatric therapies. These beneficial effects have been demonstrated for sleep disorders of different etiologies, not only primary but also associated with mental, neurologic, or other medical disorders. Regarding this issue, it is important to point out that sleep disorders of children with or without developmental disabilities are basically of circadian origin and depend on the extent and severity of brain dysfunction rather than on the cause of the condition which initiated neurological problems. Therefore, the dose of melatonin applicable to each patient must vary according to multiple factors such as the child's medical problems, the severity and type of sleep problems, or the associated neurological pathology. Interesting results have also been reported in the treatment of epilepsy, which show that melatonin reduces not only the convulsive seizures but prevents neuronal damage associated with seizures and improves sleep quality in these patients. The hypothesis of the possible role of a melatonin deficit in the etiology of adolescent idiopathic scoliosis encouraged its use and showed that it delayed the progression of the spinal scoliosis in children with low melatonin levels. In neonatal therapy, the antioxidant properties of melatonin have been the basis for its administration to newborns to reduce oxidative stress caused by sepsis, asphyxia, respiratory distress, or surgical stress. The feeding of infants with either breast or formula milk with different concentration of melatonin for the night and at day meals contributed to a better consolidation of the sleep/wake rhythm of the children. It is noteworthy that significant side effects of melatonin in children have not been reported [5]. In conclusion, there is certainly clinical evidence for the usefulness of melatonin in the treatment of some pediatric pathologies. However, the number of controlled clinical trials is still small, and specific studies to resolve problems of dosage, formulations (slow or fast release), and length of treatment are desirable.
